# Metabolically Unhealthy Phenotype: A Key Factor in Determining “Pediatric” Frailty

**DOI:** 10.3390/pediatric13030042

**Published:** 2021-07-01

**Authors:** Valeria Calcaterra, Hellas Cena, Annamaria Ruggieri, Gianvincenzo Zuccotti, Annalisa De Silvestri, Gianni Bonalumi, Gloria Pelizzo

**Affiliations:** 1 Pediatric and Adolescent Unit, Department of Internal Medicine, University of Pavia, 27100 Pavia, Italy; valeria.calcaterra@unipv.it; 2 Pediatric Unit, “V. Buzzi” Children’s Hospital, 20154 Milano, Italy; gianvincenzo.zuccotti@unimi.it; 3 Laboratory of Dietetics and Clinical Nutrition, Department of Public Health, Experimental and Forensic Medicine, University of Pavia, 27100 Pavia, Italy; hellas.cena@unipv.it; 4 Clinical Nutrition and Dietetics Service, Unit of Internal Medicine and Endocrinology, ICS Maugeri IRCCS, 27100 Pavia, Italy; 5 Vascular Surgery Unit, Istituto di Cura Città di Pavia, 27100 Pavia, Italy; annam.ruggieri@gmail.com (A.R.); bonalumi.gianni@gmail.com (G.B.); 6 Department of Biomedical and Clinical Science “L. Sacco”, University of Milano, 20154 Milano, Italy; 7 Clinical Epidemiology & Biometry, IRCCS Policlinico San Matteo Foundation, 27100 Pavia, Italy; a.desilvestri@smatteo.pv.it; 8 Pediatric Surgery Unit, “V. Buzzi” Children’s Hospital, 20154 Milano, Italy

**Keywords:** frailty, metabolic syndrome, obesity, undernutrition, children, age

## Abstract

Frailty (FI) and metabolic syndrome (MS) are each associated with adverse health outcomes. A relationship between FI and MS has previously been described in adults. We considered the prevalence of a metabolically unhealthy phenotype (MUP) in malnourished children with neurological impairment and in subjects with obesity in comparison to a group of elderly individuals at risk of FI, and we did so in order to define the potential similarities that may underline the risk of FI in specific children. We considered 50 undernourished (defined as having a body mass index of BMI ≤ 2, standard deviation score, SDS, according to World Health Organization) disabled children; 50 children with obesity (BMI ≥ 2 SDS); 50 children who were a normal weight (−1 SDS ≤ BMI ≤ +1 SDS); 21 patients who were >75 years old. MUP was defined as the presence of at least one of the following risk factors: hypertension, hyperglycemia or diabetes, hypercholesterolemia, and hypertriglyceridemia. In children with a disability and obesity, a higher prevalence (*p* < 0.001) and risk (disability OR 54.88, obesity OR 13.37) of MUP was noted compared to children of a normal weight. Compared to elderly patients, the prevalence of MUP did not differ in disabled children. On the contrary, MUP was lower in children with obesity (*p* < 0.001) and in pediatric subjects of a normal weight (*p* < 0.01). MS might play a key role in “pediatric” frailty. The extremities of the aging process and malnutrition are likely key factors in the development of FI. A multidisciplinary approach to FI may represent an important milestone for pediatric care.

## 1. Introduction

Frailty (FI) is an age-related, multidimensional condition representing an increased vulnerability of some individuals to adverse health outcomes compared to others of the same age [1,2]. FI captures unexplained heterogeneity in risk for people of the same age, and results in physiological multisystem failure, including neurological, endocrine, immune, and skeletal muscle failures [1,2].

FI is considered a result of the ageing process and it is usually described in elderly individuals. However, the concept of FI should be also considered in other patients, including pediatrics. This is important when referring to a specific subset of individuals with high cumulative biological risk, such as children with neurological impairments (NI) and children with obesity [3,4]. In these risk groups, cellular senescence may occur in response to various forms of cellular stress, and it is characterized by a pro-inflammatory secretory phenotype [5,6].

The correlation between the high risk of the components of metabolic syndrome (MS) and high cumulative biological dysregulation, also referred to allostatic load (AL), has already been described in pediatrics [4]. As for FI, MS is associated with adverse outcomes in adults, and this relationship has been acknowledged [7].

In this brief report, we considered the prevalence of a metabolically unhealthy phenotype (MUP) itself determined as being more than one component of MS in malnourished children with a severe neurological disability and in subjects with obesity in comparison to a group of elderly individuals at risk of FI. The goal was to define a potential similarity that may underline a risk for FI in selected children with high biological dysregulation, through which the ageing mechanism could occur precociously. The components of MS could represent a key factor in determining “pediatric” FI.

## 2. Patients and Methods

### 2.1. Patients

In this retrospective study, the sample included consisted of the following:

- Fifty undernourished children (defined as having a body mass index of BMI ≤ 2, standard deviation score, SDS, according to World Health Organization [8]) with severe NI (Level 5m according to Gross Motor Function Classification System [6]; cerebral palsy was apparent in 38% of subjects; 36% suffered from epileptic encephlopathy; a neurological disability in dysmorphic syndrome was clear in 26%). All NI children were bedridden and lived in sheltered communities. In all subjects, at least 2 anticonvulsive drugs were administered, including phenobarbital, phenytoin, valproic acid, topiramate, lamotrigine, carbamazepine, and clonazepam, and enteral feeding was adopted. The patients were enrolled between 1 February 2016 and 1 June 2016, and referred to the Pediatric Surgery Unit, Fondazione IRCCS Policlinico San Matteo for treatment and/or management of nutrition support.
- Fifty children with obesity (BMI ≥ 2 SDS) [8] comparable for age and sex. Due to excessive body weight, these subjects were referred by their general practitioner or primary care pediatrician to the outpatient clinic of the Pediatric Endocrinology Unit and were consecutively enrolled. Children were excluded from enrollment if they had concurrent chronic or acute illnesses, any known secondary syndromes, or were on any medication.
- Fifty historical normal weight children (−1 SDS ≤BMI ≤ +1 SDS) [8] matched for age and sex. The subjects were admitted to the pediatric outpatient clinic of the Pediatric Endocrinology Unit and were referred by their general practitioner or primary care pediatrician for auxological evaluation. They were consecutively enrolled.
- Twenty-one hospitalized patients older than 75 were included as an FI risk group. The patients were referred to a clinic (Istituto di Cura Città di Pavia) for vascular evaluation.

Anthropometric parameters, blood pressure, and biochemical parameters were considered in all enrolled subjects.

Metabolically unhealthy phenotype (MUP) was defined as the presence of at least one of the following risk factors [9,10,11]: systolic blood pressure (SBP) and/or diastolic blood pressure (DBP) > 90th percentile by gender, age, and height percentile (patients < 18 years), or SBP > 130 mmHg and/or DBP > 85 mmHg (adults); glycemia > 100 mg/dL or diabetes; hypercholesterolemia (high-density lipoprotein (HDL)-cholesterol *<* 40 mg/dL and/or total cholesterol > 200 mg/dL); TGs > 100 mg/dL (patients < 10 years) or >130 mg/dL (patients ≥ 10 years/adults).

### 2.2. Anthropometric Parameters and Blood Pressure

The physical examination of the children included an evaluation of weight, height, BMI (calculated as body weight in kilograms divided by height in meters squared), waist circumference, and waist-to-height ratio (WHtR), as well as a pubertal stage evaluation [3,4] and SBP and DBP blood pressure measurements. In adults, the height, weight, BMI, and blood pressure were recorded.

In order to measure weight in disabled subjects, patients were first weighed while being held by one of their parents/legal caregivers. Next, the parent/legal caregiver was weighed. Finally, the difference between both weights was obtained. To obtain reliable measurements of height and length, anthropometry was performed to measure the length of body segments according to Stevenson’s method [12]. Total length of the tibia was obtained by measuring the straight distance from the cranial articular surface to the fibular condyle of the tibia, i.e., lateral condyle to the tip of the medial malleolus. Ulna length was measured from the tip of the olecranon process to the tip of the styloid process. Data corresponding to the average of the ulna measurements and tibia lengths were used to obtain an estimate of stature according to specific equations [12]

In children with obesity, anthropometric measurements were performed as previously reported [4].

Blood pressure readings were taken twice using a mercury sphygmomanometer and after subjects had been sitting comfortably for 5 min. An appropriately sized cuff was used on the right arm, which was slightly flexed at heart level. The second BP measurement was used for the analysis.

### 2.3. Biochemical Parameters

Blood samples were drawn in the morning after overnight fasting. In all patients, biochemical measures included fasting blood glucose (FBG), high-density lipoprotein cholesterol (HDL-C), and/or total cholesterol triglycerides (TGs).

In children, insulin resistance (IR) was also considered a condition of pre-diabetes, at least within the parameters of the homeostasis model assessment of insulin resistance (HOMA-IR) index.

As previously described [3,4], plasma glucose was measured using the hexokinase-G-6-phosphate dehydrogenase method (Siemens Healthcare Diagnostics, Camberley, UK) with a chemistry analyzer (Advia XPT, Siemens). An enzymatic method (Advia XPT, Siemens Healthcare Diagnostics, U.K.) was used to determine total cholesterol. HDL cholesterol was measured by means of the selective detergent method, followed by enzymatic reactions (Siemens Healthcare Diagnostics). The glycerol phosphatase oxidase method was used to measure TGs concentrations (Siemens Healthcare Diagnostics, UK). Serum insulin was measured with a solid-phase, two-site chemiluminescent immunometric assay with an immunochemistry analyzer (Immulite 2000, Siemens Healthcare Diagnostics, UK). AST, ALT, and GGT were measured with a chemistry analyzer (Advia XPT, Siemens Healthcare) equipped with dedicated reagents; the transaminase assay method is based on nicotinamide adenine dinucleotide ((NAD)H monitoring by ultraviolet (UV) detection without the addition of P-5′-P. The GGT assay method is based on the transfer of the gamma-glutamyl group from L-gamma-glutamyl-3-carboxy-4-nitroaniline to the glycylglycine acceptor to yield 3-carboxy-4-nitroaniline, which is measured. Insulin resistance was determined by means of the homeostasis model assessment for insulin resistance (HOMA-IR) using the following formula: insulin resistance = (insulin × glucose)/22.5 [13].

### 2.4. Statistical Methods

Categorical variables were described by percentages and continuous variables were reported as means and standard deviations. A comparison across groups was performed by Chi-square and post-hoc *t*-test following one-way ANOVA considering 0.05 as significance level to judge *p*-values.

Power analysis reveals that for primary endpoint (comparing prevalence of MUP in malnourished children with severe neurological disability and with obesity compared to a group of elderly at risk for FI) a sample size of 120 achieves 85% power to detect the observed effect size (W) of 0.3026 using a 2 degrees of freedom Chi-Square Test with a significance level (alpha) of 0.05.

The data analysis was performed with the STATA statistical package (release 16.1, Stata Corporation, College Station, TX, USA).

## 3. Results

The characteristics of the pediatric and old patients are reported in Table 1.

### 3.1. Pediatric Groups

In disabled children, MUP prevalence was higher than in children with obesity or in those of a normal weight (*p* < 0.001).

As reported in Table 1, children with NI showed higher values of insulin, IR, TGs, and HDL-cholesterol (*p* < 0.001) compared to children of a normal weight, and significantly higher HOMA-IR compared to pediatric patients with obesity (*p* < 0.04). Higher WC, WHtR, and SBP were detected in children with obesity compared to other pediatric groups (*p* < 0.01); WHtR and DBP were higher in children with NI and obesity than in those of a normal weight (*p* < 0.001 and *p* = 0.03, respectively)

**Figure 1:**
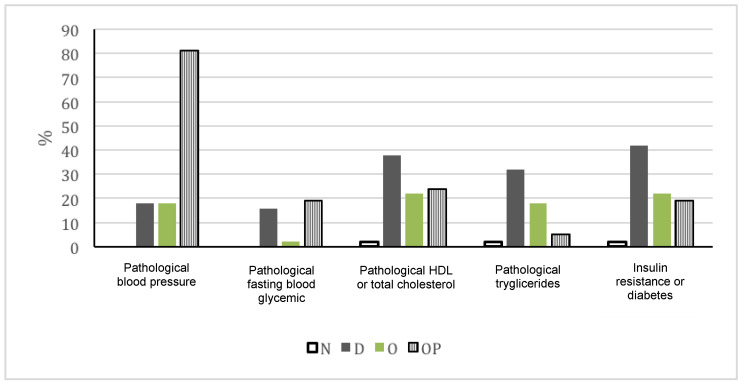
Pathological components of metabolic syndrome in pediatric groups (N = normal weight children; D = disabled patients; O = obese subjects) and older patients (OP= older patients).

As reported in Table 1 and Figure 1, the presence of pathological levels of blood pressure, cholesterol values, TGs, and IR was more frequent both in disabled patients and in children with obesity compared to normal weight patients (*p* < 0.01), even without there being a difference between the disability and obesity subgroups (*p* > 0.05). Pathological FBG was more prevalent in disabled children than in children with obesity and normal-weight ones (*p* < 0.01).

Compared to children of a normal weight, MUP risk was significantly higher in children with neurological impairment and obesity (*p* < 0.001; disabled children OR 54.88 (95%CI 14.03–214.6); children with obesity OR 13.37 (95%CI 3.66–48.82)), with a higher risk in disabled children (*p* = 0.002).

### 3.2. Pediatric Groups in Comparison to Older Patients

MUP prevalence in disabled children (*p* = 0.45) did not differ from that in the elderly group. On the contrary, MUP was lower in children with obesity (*p* < 0.01) and in normal weight pediatric subjects (*p* < 0.001) (Table 1).

The prevalence of pathological blood pressure values was higher in the elderly group compared to all pediatric groups (*p* < 0.001) (Table 1 and Figure 1). The prevalence of pathological FBG was higher in disabled children compared to other pediatric groups (*p* < 0.001). Pathological TGs levels were reported more often in disabled children compared to the elderly group (*p* = 0.01). The prevalence of IR e/o diabetes in the elderly group was not significantly different compared to what was observed in children with a disability (*p* = 0.06) or in those with obesity (*p* = 0.078).

## 4. Discussion

Frailty is characterized by functional decline across multiple inter-related systems, such as the organs and homeostatic reserves, as well as a resistance to stressors, which could cause vulnerability to adverse health outcomes [1,2]. The process of vulnerability and decline is inextricably linked to the aging process [14,15]. However, certain pediatric conditions, such as disability and obesity, may share some of the same characteristics as FI.

Neurologically disabled children are particularly vulnerable to undernutrition and tend to be at risk of comorbidities, including metabolic disorders and AL, which contribute to cardiovascular disease [16]. Their multiorgan involvement is considered a risk factor for increased morbidity and mortality compared to healthy children.

Similarly to disability, excessive body weight is associated with different sequelae, including dismetabolic conditions, diabetes mellitus, cardiovascular disease, increased risk for malignancies, or musculoskeletal disorders. As reported in a previous manuscript of ours, obesity-related comorbidities start early in childhood, increasing the risk of premature death [7].

The multisystem damage, homeostatic dysregulation, and vulnerability to adverse health outcomes reported in disability and obesity have all shown that the development of FI characteristics is an inevitable outcome of these conditions [1,2].

The physiopathogenetic mechanisms of FI syndrome are not completely understood, but they may include chronic low-grade inflammation, prothrombotic state, oxidation, and increased IR and dehydration [17].

The elderly are vulnerable to dehydration due to physiological changes in the ageing process, but this can be complicated by many disease states. Moreover, a loss of thirst and a decreased ability to concentrate urine can lead to hyperosmotic stress, with consequent cell dehydration and cell damage [17]. Dehydration is common and frequently under-diagnosed in chronically malnourished children who are affected by different forms of disability [9]; it has also been found to be significantly associated with elevated BMI on a population level [18].

Other than dehydration, all the biological processes we mentioned previously are also altered in individuals with MS.

As reported in the literature and confirmed in our study, malnourished children with NI and obesity showed a higher prevalence of MUP compared to age- and sex-matched children, with no difference in disabled children compared to the elderly group. The results support the hypothesis that MS could be a key factor in determining “pediatric” frailty. This condition represents an early step in the disablement process that is potentially reversible [1,2,10,11]. In fact, given that MS may be controlled or prevented, our results emphasize the need for a specific intervention for vulnerable children that focuses on healthy behaviors in order to limit the detrimental health impacts of MS.

Children and the elderly represent opposite ends of the human lifespan, confirming the link between frailty and the aging process. On the other hand, under-nutrition and obesity represent the opposite ends of malnutrition, supporting the suggestion that nutritional status plays a key role in FI development, which is well acknowledged in the aging process.

We recognize that this study has some limitations. First, it is a retrospective study and results may be inferred by some bias. Second, the sample size is small and therefore not representative of the whole disabled population or of the pediatric population affected by obesity. Additionally, a lack of consensus regarding the definition of FI at pediatric age and the FI index in the elderly enrolled sample does not allow for a precise classification based on FI in these subjects.

However, the prevalence rate of MUP observed both in children with a disability and in those with obesity, as well as in the elderly subgroup compared to controls, suggests that components of MS may be key factors in determining “pediatric” frailty. A relationship between MUP and FI cannot be excluded in relation to pediatric age. The opposite ends of the aging process and malnutrition play a role in the concept of FI, for which prevention is imperative.

A multidisciplinary approach to FI may represent an important milestone for pediatric clinical care, providing accessible healthcare for at least a specific subset of children.

## 5. Conclusions

MS might play a key role in “pediatric” frailty. Extremities of the aging process and malnutrition are likely key factors in the development of FI. A multidisciplinary approach to FI may represent an important milestone for pediatric care.

## Figures and Tables

**Table 1:** Clinical, metabolic and cardiovascular parameters in pediatric and old patients.

Variables	Pediatric Patients			Old Patients (OP)	*p* Value					
	Normal Weight (N)	Disabled (D)	Obese (O)		N vs. D	N vs. O	O vs. D	N vs. OP	OP vs. D	O vs. OP
Age (years)	11.97 (3.59)	11.82 (5.82)	11.51 (3.18)	79.2 (3,2)	0.88	0.50	0.73	<0.001	<0.001	<0.001
Females *n* (%)	23 (46)	22 (44)	25 (50)	10 (47.62)	0.84	0.68	0.54	0.90	0.776	0.85
Metabolically unhealthy phenotype *n* (%)	3 (6)	35 (77.78)	23 (46)	18 (85.71)	<0.001	<0.01	<0.001	<0.001	0.45	<0.01
Waist circumference (cm)	67.75 (8.91)	67.23 (15.4)	84.41 (8.98)	nd	0.83	<0.001	<0.001	nd	nd	nd
Waist to height ratio	0.48 (0.14)	0.5 (0.11)	0.56 (0.6)	nd	<0.001	<0.001	<0.001			
Glycemia (mg/dL)	77.36 (9.42)	82.24 (48.31)	72.78 (11.9)	99.57 (26.72)	0.48	0.03	0.18	<0.001	0.13	<0.001
Fasting insulin (μUI/mL)	8.4 (2.8–11.6)	14.5 (5.5–24.35)	9.7 (6.1–15.6)		<0.001	0.02	0.06	nd	nd	nd
HOMA-IR	1.53 (.45–2	2.75 (1.06–5)	1.544 (1.05–2)		<0.001	0.07	0.04	nd	nd	nd
Tryglicerides (mg/dL)	63.5 (49.5–87)	85 (66–131)	73 (59–104)	92 (77–129)	<0.001	0.01	0.12	<0.001	0.31	0.11
Total cholesterol (mg/dL)	151.08 (24.02)	146.02 (36.83)	159.84 (30.96)	169.81 (52.94)	0.53	0.21	0.05	0.11	0.03	0.32
HDL cholesterol (mg/dL)	54.67 (10.41)	44.07 (13.07)	47.6 (10.1)		<0.001	<0.001	0.14	nd	nd	nd
Systolic blood pressure (mmHg)	105.4 (9.03)	103.6 (17.39)	113.76 (10.22)	138.71 (16.44)	0.52	<0.01	<0.001	<0.001	<0.001	<0.001
Diastolic blood pressure (mmHg)	67 (8.39)	65.91 (12.9)	70.64 (8.3)	82.29 (7.38)	0.62	0.03	0.03	<0.001	<0.001	<0.001
GOT (U/L)	23.3 (6.42)	26.53 (13.32)	22.85 (6.44)	19.48 (7.03)	0.22	0.76	0.09	0.04	0,02	0.05
GPT (U/L)	14 (12–16)	14.5 (11.5–22.5)	15 (13–24)	77 (62–90)	0.49	0.07	0.53	<0.001	<0.001	<0.001
GGT (U/L)	12.85 (5.15)	28.21 (21.06)	15.6 (6.74)	29 (21)	<0.001	0.07	0.03	<0.001	0.88	<0.001
Pathological blood pressure *n* (%)	0 (0)	9 (18)	9 (18)	17 (80.95)	<0.01	0.001	1	<0.001	<0.001	<0.001
Pathological fasting blood glycemia *n* (%)	0 (0)	7 (15.56)	1 (2)	4 (19.05)	<0.01	0.31	0.01	<0.01	0.72	0.01
Pathological HDL or total cholesterol *n* (%)	1 (2)	17 (37.78)	11 (22)	5 (23.81)	<0.001	<0.01	0.09	<0.01	0.26	0.86
Pathological tryglicerides *n* (%)	1 (2)	16 (32)	9 (18)	1 (4.76)	<0.001	<0.01	0.10	0.52	0.01	0.14
Insulin resistance or diabetes *n* (%)	1 (2)	21 (42)	11 (22)	4 (19.05)	<0.001	<0.01	0.03	0.01	0.06	0.78

HOMA-IR: Homeostatic Model Assessment for Insulin Resistance; HDL: high-density lipoprotein; GOT: glutamic oxaloacetic transaminase; GPT GGT.

## Data Availability

The data are contained within the article.
